# Accumulation of saturated intramyocellular lipid is associated with insulin resistance[S]

**DOI:** 10.1194/jlr.M091942

**Published:** 2019-05-02

**Authors:** David B. Savage, Laura Watson, Katie Carr, Claire Adams, Soren Brage, Krishna K. Chatterjee, Leanne Hodson, Chris Boesch, Graham J. Kemp, Alison Sleigh

**Affiliations:** Metabolic Research Laboratories, Wellcome Trust-MRC Institute of Metabolic Science,* Cambridge Biomedical Campus, Cambridge, United Kingdom; National Institute for Health Research/Wellcome Trust Clinical Research Facility, Cambridge University Hospitals NHS Foundation Trust† Cambridge Biomedical Campus, Cambridge, United Kingdom; Oxford Centre for Diabetes, Endocrinology and Metabolism,** Radcliffe Department of Medicine, University of Oxford, Oxford, United Kingdom; Department of Clinical Research and Radiology†† AMSM, University Bern, Bern, Switzerland; Department of Musculoskeletal Biology§§ University of Liverpool and MRC–Arthritis Research UK Centre for Integrated Research into Musculoskeletal Ageing, Liverpool, United Kingdom; Wolfson Brain Imaging Centre*** University of Cambridge School of Clinical Medicine, Cambridge, United Kingdom; MRC Epidemiology Unit§ University of Cambridge School of Clinical Medicine, Cambridge, United Kingdom

**Keywords:** triglycerides, fatty acids, lipodystrophies, spectroscopy, lipid composition, muscle, exercise, in vivo

## Abstract

Intramyocellular lipid (IMCL) accumulation has been linked to both insulin-resistant and insulin-sensitive (athletes) states. Biochemical analysis of intramuscular triglyceride composition is confounded by extramyocellular triglycerides in biopsy samples, and hence the specific composition of IMCLs is unknown in these states. ^1^H magnetic resonance spectroscopy (MRS) can be used to overcome this problem. Thus, we used a recently validated ^1^H MRS method to compare the compositional saturation index (CH_2_:CH_3_) and concentration independent of the composition (CH_3_) of IMCLs in the soleus and tibialis anterior muscles of 16 female insulin-resistant lipodystrophic subjects with that of age- and gender-matched athletes (*n* = 14) and healthy controls (*n* = 41). The IMCL CH_2_:CH_3_ ratio was significantly higher in both muscles of the lipodystrophic subjects compared with controls but was similar in athletes and controls. IMCL CH_2_:CH_3_ was dependent on the IMCL concentration in the controls and, after adjusting the compositional index for quantity (CH_2_:CH_3adj_), could distinguish lipodystrophics from athletes. This CH_2_:CH_3adj_ marker had a stronger relationship with insulin resistance than IMCL concentration alone and was inversely related to VO_2max_. The association of insulin resistance with the accumulation of saturated IMCLs is consistent with a potential pathogenic role for saturated fat and the reported benefits of exercise and diet in insulin-resistant states.

After it was demonstrated that ^1^H magnetic resonance spectroscopy (MRS) can noninvasively distinguish intramyocellular lipids (IMCLs) from extramyocellular lipids (EMCLs) (1, 2), associations were reported between soleus (SOL) IMCL accumulation and insulin resistance independent of fat mass (3–5). Given that skeletal muscle represents the primary site for postprandial glucose disposal (6), these findings were of considerable physiological interest. Furthermore, these data strongly supported the link between ectopic fat accumulation and insulin resistance (7, 8). Although it soon became clear that triglycerides themselves were unlikely to be involved in causing insulin resistance, intramuscular triglyceride content does seem to correlate with insulin resistance in some states (5, 9–12). One particularly striking and surprising exception was reported in athletes, in which histological studies suggested that neutral lipid accumulation was a feature of skeletal muscle in insulin-sensitive, endurance-trained athletes (13, 14), and this finding has led to the now widely cited notion of an “athlete’s paradox” (13). This concept is consistent with the idea that triglyceride content itself is not casually involved in insulin resistance and has prompted several efforts to identify the lipid intermediates responsible for causing insulin resistance or preserving the insulin sensitivity of athletes.

Saturated fat has been implicated in the pathogenesis of metabolic disease (15, 16), and we have recently described and validated (using IMCL/EMCL-simulated phantoms of known composition) a ^1^H MRS method that provides an in vivo compositional marker of IMCLs that primarily reflects the degree of saturation of the FA chains within triglycerides (17). This marker, which we call the IMCL saturation index (CH_2_:CH_3_), utilizes good-quality spectra acquired at 3T with a short echo time and compares the CH_2_ resonance located at 1.3 ppm (which is influenced by both concentration and composition) with that of the CH_3_ resonance at 0.9 ppm (which is independent of triglyceride composition); this is illustrated in **Fig. 1**. Figure 1 also shows that using a concentration of hydrogen that resonates at 1.3 ppm (CH_2_) to represent the concentration of lipids without knowing the underlying composition, as has been the practice in virtually all published ^1^H MRS studies of IMCLs so far, confounds the contributions of both the concentration of lipids and their composition. This can potentially lead to a significant error in estimating the concentration: the composition would contribute as much as 50% to the observed signal (equivalent to a 100% theoretical increase in the signal) if the pool were stearic acid instead of linoleic acid. Therefore, we used the IMCL CH_3_ peak at 0.9 ppm to estimate the total concentration of IMCLs, as this is independent of the degree of saturation of the FA chains within triglycerides (i.e., composition). We call this the composition-independent IMCL concentration estimate to distinguish it from the conventional estimate using CH_2_.

**Fig. 1.**
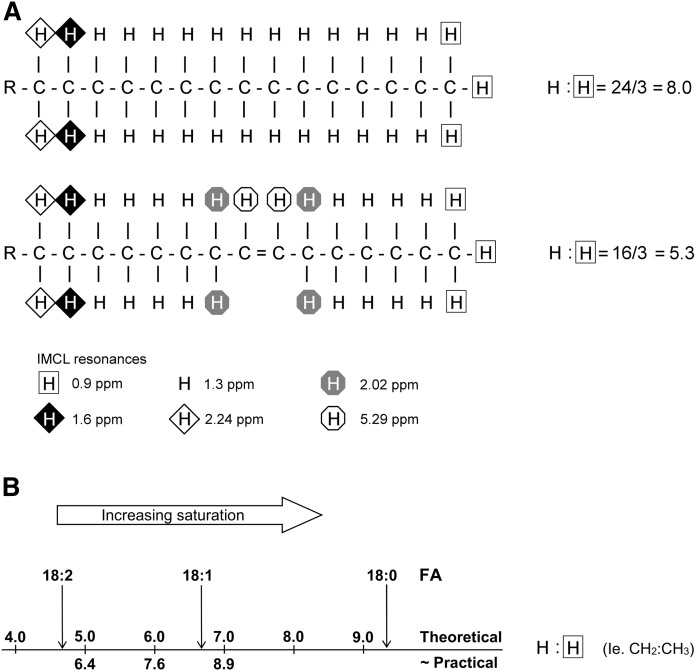
The ratio of CH_2_:CH_3_ is influenced primarily by the degree of saturation of the FAs within triglycerides. This figure shows the principle of the CH_2_:CH_3_ ratio as a compositional saturation index of IMCLs. A: In this example, the palmitoleic acid component of triglyceride has a theoretical ratio of CH_2_ (at 1.3 ppm) to CH_3_ (at 0.9 ppm) of 16/3 = 5.3, which is lower than the equivalent ratio of palmitic acid (8.0). This is due not only to the desaturation of two CH_2_ groups but also to the alteration in the chemical environment of the neighboring CH_2_ groups. For FA chains with an equal number of double bonds, the CH_2_:CH_3_ ratio will also be scaled by chain length, although this will have a proportionally smaller effect (17). B: Theoretical CH_2_:CH_3_ values for stearic, oleic, and linoleic FAs and approximate practical values, which are systematically shifted with respect to the theoretical values, as discussed in Thankamony et al. (17). Part of this figure has been reproduced from Thankamony et al. (17), which is licensed under a Creative Commons Attribution 4.0 International License.

Lipodystrophy is a rare cause of severe insulin resistance and is typically characterized by prominent ectopic fat accumulation due to both the reduction in adipocyte lipid storage capacity and the associated hyperphagia induced by leptin deficiency. To ascertain whether IMCL composition is altered in lipodystrophic (LD) subjects and if such changes in lipid composition might help to elucidate the athlete’s paradox, we determined the compositional saturation index (CH_2_:CH_3_ ratio) and composition-independent concentration (from CH_3_) of IMCLs in the SOL and tibialis anterior (TA) muscles of female insulin-resistant LD subjects as well as age- and gender-matched athletes and nonathlete controls.

## MATERIALS AND METHODS

### Participants

Sixteen female subjects with lipodystrophy were identified as part of a long-standing study of human insulin-resistant syndromes, while age- and gender-matched controls (*n* = 41) and athletes (*n* = 14) were recruited by advertisement. SOL IMCL data from five of the subjects were included in a previously published study (18). Control and athlete exclusion criteria included current smoking; drug or alcohol addiction; any current or past medical disorder or medications that could affect measurements, including supplements such as creatine; and standard MRI contraindications. Controls were recruited who exercised less than three times per week for 1 h each time, while the athletes, some of whom competed in international events, were part of a running club and regularly ran distances between 10 and 40 km. Subjects with lipodystrophy were recruited if they could perform an overnight fast without insulin or a rapid-acting insulin analogue. The studies relating to lipodystrophy were approved by the NHS Research Ethics Committee, and the healthy volunteer studies were approved by the East of England Cambridge Central Ethics Committee. Studies were conducted in accordance with the Declaration of Helsinki, and all participants provided written informed consent.

### Protocol

Volunteers were instructed to follow normal dietary habits for 3 days before arriving at the National Institute for Health Research (NIHR)/Wellcome Trust Clinical Research Facility. Participants provided fasting blood samples and were given a light breakfast of either toast or cereal immediately prior to ^1^H MRS. Athletes and controls were instructed to refrain from vigorous exercise for at least 24 and 19 h, respectively, prior to ^1^H MRS.

HOMA-IR was calculated as fasting insulin (pmol/l) × fasting glucose (μU/ml)/22.5. Body composition was assessed by dual-energy X-ray absorptiometry (Lunar Prodigy enCORE version 12.5 for controls and Lunar iDXA enCORE version 16 for athletes; GE Healthcare, Madison, WI).

### ^1^H MRS

^1^H MRS studies were performed on a 3T scanner (Siemens; Erlangen, Germany) using the point-resolved spectroscopy sequence with a short echo time of 35 ms. A water-suppressed ^1^H spectrum was acquired from a voxel with a cube length of 1.3 cm positioned to avoid visible fat on T_1_-weighted images within TA and SOL using a 5 s repetition time and 64 averages (4 averages for the nonwater-suppressed spectrum). Data were analyzed in jMRUI (19, 20) and fitted with the AMARES (21) algorithm using identical prior-knowledge parameters: Gaussian line shapes (except water: Lorentzian), soft constraints on EMCL/IMCL CH_2_ frequencies and line widths, CH_3_ resonant frequencies and line widths determined from known and inferred prior knowledge relative to the CH_2_ resonance (22), and with all amplitudes estimated. Because the CH_3_ resonance is small and may be subject to spectral overlap, the results were later checked for robustness by reanalyzing the data using different fitting parameters, as outlined in supplemental Table S1. IMCL CH_2_ and CH_3_ are quantified relative to the methyl group of creatine plus phosphocreatine at 3.0 ppm. Because this resonance exhibits different line-shape characteristics in the TA and SOL muscles (23), comparable quantification between muscles using a nominal concentration of muscle creatine is not valid; instead, a scaling factor of creatine to water for each muscle was established from a subset of participants who had nonwater-suppressed data sets, yielding a calculated water signal. Absolute composition-independent IMCL concentrations in mmol/kg muscle wet weight were calculated from the compositionally invariant CH_3_ IMCL resonance, with standard assumptions regarding muscle water content and correction for T_2_ relaxation effects, J coupling, and proton density as outlined below.

Absolute IMCL concentrations in mmol/kg muscle wet weight were calculated using the CH_3_ IMCL resonance (which is compositionally invariant) using the following equation:

$$\left[\text{IMCL}\right]=\left({\text{S}}_{\text{o IMCL CH}3}/{\text{S}}_{\text{o water}-\text{calc}}\right).\left[\text{water}\right]$$

where *S*_o_ is the corrected signal intensity of the resonance, water-calc is the calculated water signal from the internal standard (creatine and phosphocreatine), and [water] is the concentration of water in skeletal muscle [calculated using a pure water concentration of 55,342 mmol/l and assuming a relative tissue water content in human skeletal muscle of 0.81 (kg/kg) and tissue density of 1.05 g/ml (24)]. Because the IMCL CH_3_ resonance is subject to J-coupling effects and has an unknown T_2_ relaxation time, we utilized the theoretical-to-measured IMCL CH_3_:CH_2_ ratio that would take into effect both J-coupling and T_2_ effects at this echo time as well as any bias of constrained fitting prior knowledge of the CH_3_ resonance.

$$\left({\text{S}}_{\text{o IMCL CH}3}/{\text{S}}_{\text{o water}-\text{calc}}\right)=\left({\text{S}}_{\text{o IMCL CH}3}/{\text{S}}_{\text{o IMCL CH}2}\right).\;\left({\text{S}}_{\text{o IMCL CH}2}/{\text{S}}_{\text{o water}-\text{calc}}\right)$$

Therefore,

$$\begin{array}{c} \left({\text{S}}_{\text{o IMCL CH}3}/{\text{S}}_{\text{o water}-\text{calc}}\right)=\left({\text{S}}_{\text{IMCL CH}3}/{\text{S}}_{\text{water}-\text{calc}}\right).\left(G_{\text{TMCH}3:\text{CH}2}\right).\\ \;\;\left(T_{2\text{corr}CH2/\text{water}}\right).\left({\text{n}}_{\text{water:IMCL CH}3}\right) \end{array}$$

where *S* is the uncorrected signal intensity of the resonance. (*G*_TM CH3:CH2_) = 1.1966 and is the gradient of the line of best fit through the origin of the graph of theoretical-to-measured CH_3_:CH_2_ in vitro in IMCL- and EMCL-simulated phantoms using the point-resolved spectroscopy sequence at 3T with an echo time of 35 ms with the same fitting (17), assuming that J-coupling and T_2_ relaxation effects would be similar in vivo. (*T*_2corr CH2/water_) is the correction factor for T_2_ effects of CH_2_ and water that was calculated using accepted T_2_ values at 3T for each muscle (25), and (*n*_water:IMCL CH3_) is the correction for proton density.

The IMCL saturation index (CH_2_:CH_3_) was calculated as IMCL CH_2_:CH_3_, and the IMCL saturation index adjusted for quantity (CH_2_:CH_3adj_) = CH_2_ – (*m*CH_3_ + *c*), where *m* and *c* are the gradient and intercept, respectively, of the regression line through the control data points of CH_2_ versus CH_3_. Investigators were blind to the insulin-resistance status of the participants during ^1^H MRS analysis.

### Assessment of VO_2max_

Participants underwent continuous incremental exercise testing to an 85% age-predicted maximum heart rate (controls) or volitional exhaustion (athletes) on a Trackmaster TMX425 treadmill (Med-Electronics, Beltsville, MD). LD subjects did not perform an exercise test. Oxygen consumption was measured using a spiroergometer (Medical Graphics UK Ltd, Gloucester, UK) and BreezeSuite gas-exchange software. For the control participants, a standard incremental protocol was performed (26), whereas the athletes undertook a protocol that began with a 10 min warm-up period at each participant’s preferred warm-up running speed, after which the test was initiated at 9 km/h and increased steadily (0.74 km/h/min), with a ramp at 5 min (increasing 0.5% every 15 s) until exhaustion or a plateau in VO_2_ was apparent. VO_2max_ was calculated in the control participants by extrapolating the submaximal heart rate – VO_2_ relationship to the age-predicted maximum heart rate (27).

### Statistics

All statistics were performed in SPSS Statistics 24 (IBM, Armonk, NY) with significance set at *P* < 0.05. Normality was assessed by the Shapiro-Wilk test, and nonnormally distributed data were log-transformed prior to statistical testing. ANOVA with Games-Howell post hoc analysis was used to compare means between groups, and Pearson’s correlation coefficient was used for analyzing associations. Due to the nonnormality of ln(HOMA-IR), IMCL associations with HOMA-IR were assessed by Spearman’s rank correlation coefficient. Data are presented as means ± SEMs.

## RESULTS

### Participants

**Fig. 2.**
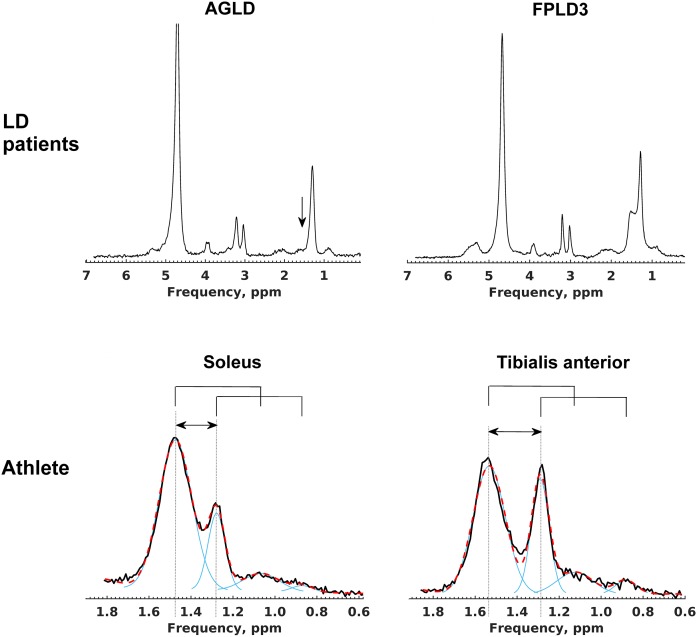
Representative ^1^H MRS spectra from LD subjects and an athlete. Water-suppressed spectra from the SOL muscle of a subject with AGLD (upper left) and FPLD3 (upper right); the absence of EMCLs in AGLD is highlighted by the vertical arrow. Spectra from an athlete’s SOL (lower left) and TA (lower right) muscles illustrate the raw data (solid black) and overall fit (red dashed) and individual fit components (solid blue) in the frequency range that contains the EMCL CH_2_ (∼1.5 ppm), CH_3_ (∼1.1 ppm), IMCL CH_2_ (1.3 ppm), and CH_3_ (0.9 ppm) resonances. The horizontal arrows indicate that the EMCL resonances are systematically shifted very slightly upfield in the SOL muscle compared with the TA due to fiber-orientation effects. The CH_3_ resonant frequencies are linked to the CH_2_ frequencies (solid bridge lines above spectra) and are also shifted. The fitting procedure fixes the relative CH_2_ to the CH_3_ frequency shift for both EMCLs and IMCLs and the CH_3_ line width relative to the corresponding CH_2_ line width but permits soft constraints on the CH_2_ frequencies.

Of the insulin-resistant subjects with lipodystrophy, 13 had partial forms [8 subjects with familial partial lipodystrophy (FPLD) type 2 due to *LMNA* mutations, 5 subjects with FPLD3 due to *PPARG* mutations), and 3 had generalized lipodystrophy [2 subjects with acquired generalized lipodystrophy (AGLD) and 1 due to mutations in the *PCYT1A* gene (28)]. Of the LD subjects, two were taking no medication at all, eight were prescribed metformin, three were taking statins, five were taking fibrates, and five were taking long-acting insulin analogues. The age- and gender-matched controls had a wide BMI range (19.6–35.6 kg/m^2^) and HOMA-IR (0.3–4.9). As a group, insulin and HOMA-IR were significantly higher in the LD subjects and lower in the athletes (**Table 1**) compared with controls, as expected. Fat mass and percentage body fat were similar between LD subjects and athletes, which were both lower compared with controls (Table 1). Serum triglycerides were higher and HDL-cholesterol concentrations were lower in the LD subjects (Table 1) compared with either controls or athletes. EMCLs were absent in the two subjects with AGLD (**Fig. 2**), but those with partial forms of lipodystrophy had an EMCL concentration such that overall LD subjects’ EMCL concentration was similar to both controls and athletes (Table 1).

**TABLE 1.** Participant characteristics and conventional muscle lipid estimates

	LD Females (*n* = 16)	Female Controls (*n* = 41)	Female Athletes (*n* = 14)	*P*			
				ANOVA	LD—Controls	LD—Athletes	Controls—Athletes
Participant characteristics							
Age (years)	38.9 ± 3.9	35.5 ± 2.0	36.5 ± 2.9	0.694			
BMI (kg/m^2^)	24.2 ± 0.7	24.4 ± 0.6	20.3 ± 0.6	**<0.001**	0.999	**0.001**	**<0.001**
Mass (kg)	66.1 ± 2.8	65.2 ± 2.3	54.6 ± 1.3	**0.013**	0.916	**0.006**	**0.001**
Fat mass (kg)	10.6 ± 1.4	23.1 ± 1.6	11.6 ± 1.1	**<0.001**	**0.001**	0.631	**<0.001**
Fat-free mass (kg)	55.4 ± 1.7	42.1 ± 0.9	43.0 ± 1.2	**<0.001**	**<0.001**	**<0.001**	0.705
Body fat (%)	16.1 ± 1.7	34.2 ± 1.2	21.1 ± 1.8	**<0.001**	**<0.001**	0.129	**<0.001**
Triglycerides (mmol/l)	3.54 ± 0.67	0.92 ± 0.07*^a^*	0.84 ± 0.08	**<0.001**	**<0.001**	**<0.001**	0.920
HDL-cholesterol (mmol/l)	0.97 ± 0.71	1.64 ± 0.06*^a^*	2.20 ± 0.12	**<0.001**	**<0.001**	**<0.001**	**0.001**
Glucose (mmol/l)	6.00 ± 0.58	4.56 ± 0.06*^a^*	4.46 ± 0.13	**<0.001**	0.063	**0.002**	0.764
Insulin (pmol/l)*^b^*	145.6 ± 22.0	38.6 ± 4.2*^a^*	22.6 ± 4.7	**<0.001**	**<0.001**	**<0.001**	**0.025**
HOMA-IR	5.53 ± 1.0	1.14 ± 0.13*^a^*	0.66 ± 0.15	**<0.001**	**<0.001**	**<0.001**	**0.025**
HbA1c (%)	6.6 ± 0.4*^c^*	ND	5.3 ± 0.1	ND		**0.008**	
VO_2max_ (ml/kg/min)	ND	36.1 ± 1.5*^d^*	46.9 ± 1.4	ND			**<0.001**
Conventional estimates*^e^*							
SOL IMCLs	1.90 ± 0.21	1.22 ± 0.07	0.79 ± 0.10	**<0.001**	**0.027**	**<0.001**	**0.007**
TA IMCLs	0.89 ± 0.16*^f^*	0.61 ± 0.04	0.45 ± 0.04	**0.035**	0.562	0.115	0.078
SOL EMCLs	2.05 ± 0.34	2.22 ± 0.19	1.81 ± 0.20	0.493			
TA EMCLs	1.43 ± 0.28*^f^*	2.30 ± 0.19	1.24 ± 0.15	**0.002**	0.163	0.785	**0.005**

Data are presented as mean ± SEM unless otherwise stated. Nonnormally distributed variables were log-transformed prior to performing ANOVA and Games-Howell post hoc analysis; bold *P* values are statistically significant.

a *n* = 38.

b To convert to μU/ml divide by 6.945.

c *n* = 13.

d *n* = 24.

e Expressed as methylene protons resonating at 1.3 ppm quantified as a percentage of the uncorrected calculated water resonance.

f *n* = 12.

### ^1^H MRS analysis of IMCL concentration

**Fig. 3.**
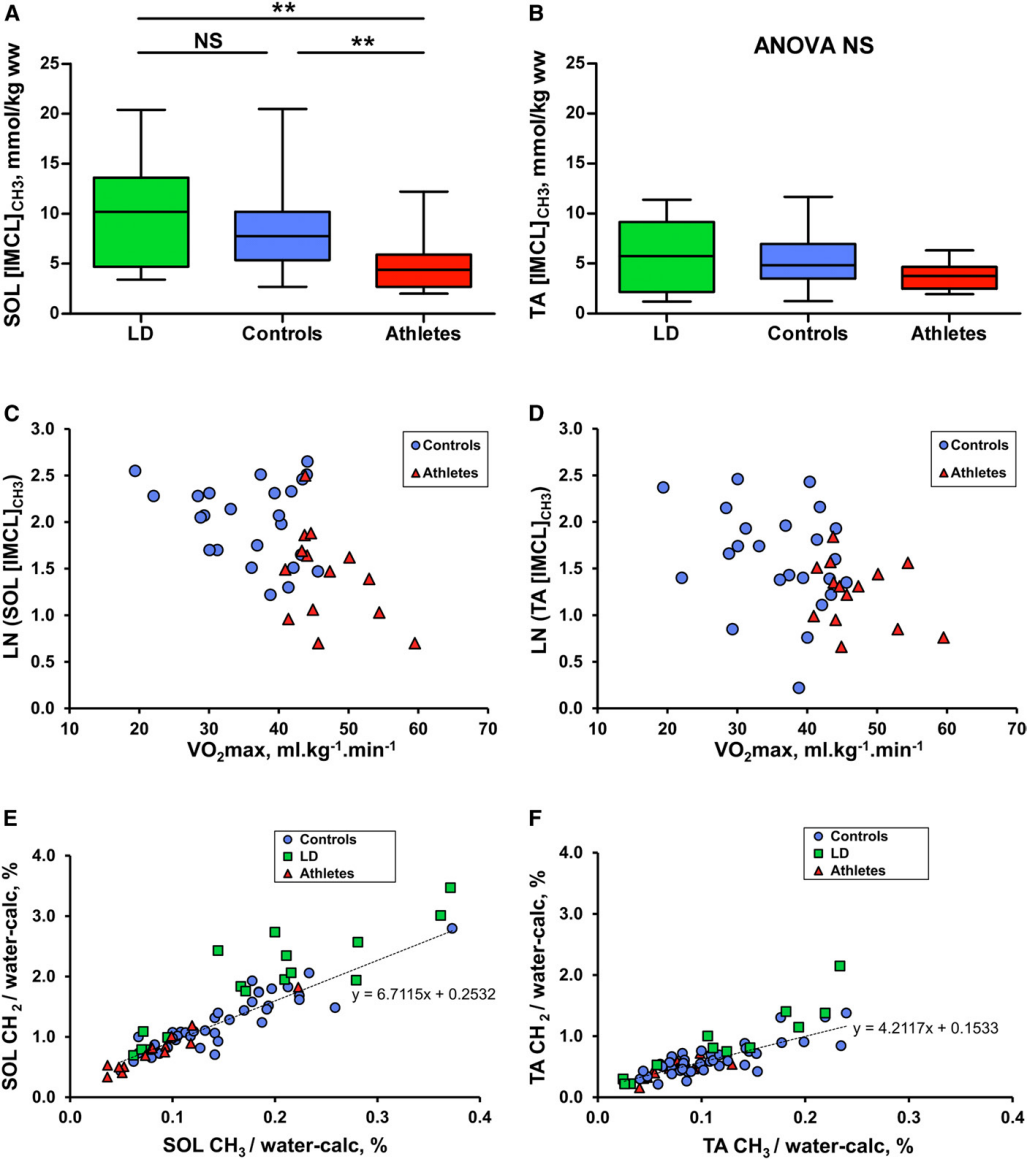
Composition-independent concentrations of IMCLs in LD subjects (green), controls (blue), and athletes (red). A, B: Box and whisker plots showing SOL and TA composition-independent IMCL concentration from the ^1^H MRS of methyl protons, as assessed by ANOVA and Games-Howell post hoc analyses. C, D: The relationship of SOL and TA composition-independent IMCL concentration with VO_2max_ in a subset of participants who underwent VO_2max_ testing, as assessed by Pearson’s correlation coefficient (controls: blue circles, *n* = 24; athletes: red triangles, *n* = 14). This was only significant when controls and athletes were combined (SOL: *r* = −0.52, *P* = 0.001; TA: *r* = −0.42, *P* = 0.009). E, F: SOL and TA IMCL CH_2_ and CH_3_ components. The values are expressed relative to the calculated water signal (water-calc), as described in Materials and Methods. The dotted line represents the linear regression line of the control data points. *P < 0.05, **P < 0.01, and ***P < 0.001.

In the SOL muscle, IMCL concentrations derived from the IMCL CH_3_ peak (0.9 ppm) (composition-independent IMCL concentrations) were not significantly increased (*P* = 0.477) in the LD subjects compared with controls but were higher compared with the lean athletes (*P* = 0.003) (**Fig. 3A**). In the more glycolytic TA muscle, composition-independent IMCL concentrations were similar in all three groups (Fig. 3B). We also observed linear inverse correlations of VO_2max_ and the IMCL concentration in the subset of controls who underwent VO_2max_ testing and athletes together (Fig. 3C, D). SOL IMCL concentration was significantly lower in the athletes compared with the controls (*P* = 0.004; Fig. 3A), and this remained significant (*P* = 0.025) compared with a subset of the controls matched for percentage body fat (controls: body fat = 21.5 ± 2.3%, *n* = 10; athletes: 21.1 ± 1.8%, *n* = 14; see supplemental Fig. S1).

The conventional estimate of IMCL concentration using the CH_2_ resonance uncorrected for composition, CH_2_/water, showed similar trends to the composition-independent estimate using the CH_3_ peak with the exception that the LD subjects’ SOL IMCL CH_2_ was significantly increased compared with controls (Table 1). This could be regarded as an artifact of the effects of compositional differences, which we consider next.

### ^1^H MRS analysis of IMCL composition

**Fig. 4.**
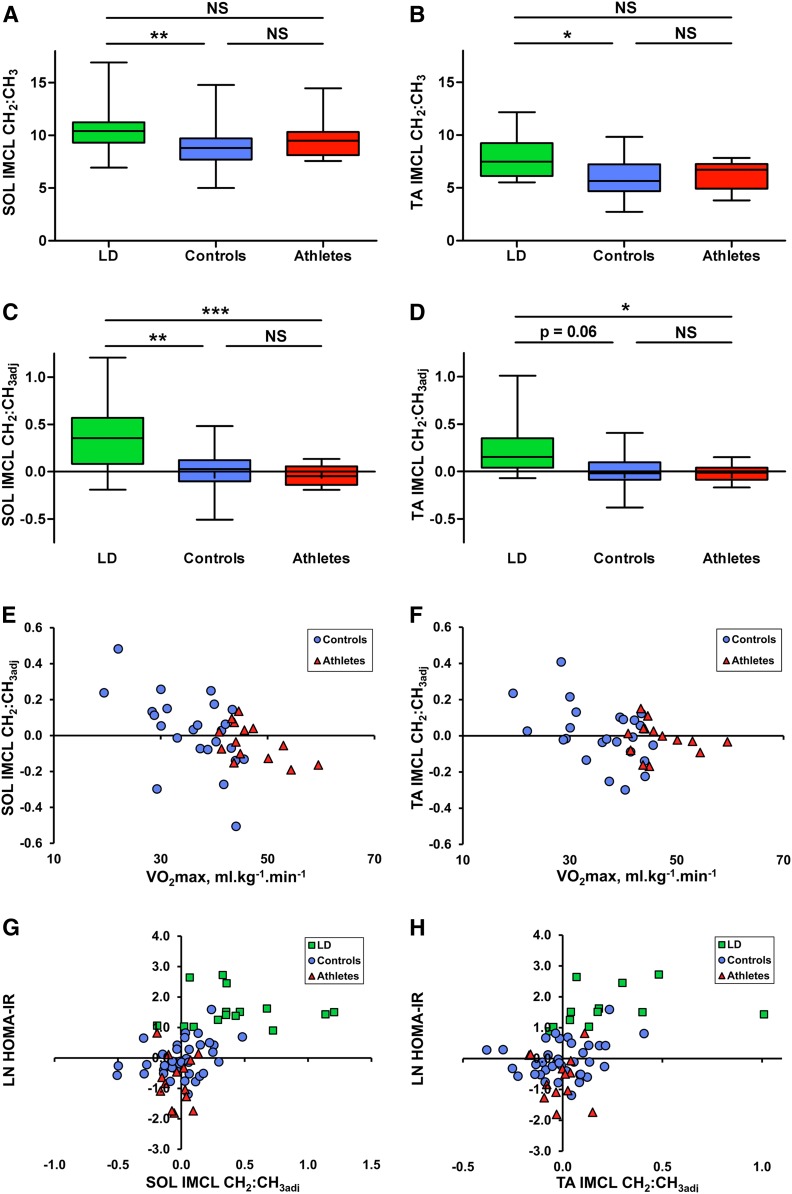
^1^H MRS measures of IMCL composition in LD subjects (green), controls (blue), and athletes (red). A, B: Box and whisker plots of (A) SOL and (B) TA IMCL compositional saturation index (CH_2_:CH_3_ ratio), as assessed by ANOVA and Games-Howell post hoc analyses. C, D: SOL and TA IMCL compositional saturation index adjusted for quantity. E, F: Relation of SOL and TA IMCL compositional adjusted saturation index with VO_2max_ in the subset of participants who underwent VO_2max_ testing, as assessed by Pearson’s correlation coefficient (controls: blue circles, *n* = 24; athletes: red triangles, *n* = 14). There was a significant correlation in the SOL (*r* = −0.546, *P* = 0.006) and TA (*r* = −0.453, *P* = 0.026) for controls alone, in the SOL (*r* = −0.558, *P* = 0.038) for athletes alone, and in the SOL (*r* = −0.520, *P* = 0.001) and TA (*r* = −0.362, *P* = 0.025) for controls and athletes combined. G, H: Relation of HOMA-IR with SOL and TA IMCL compositional adjusted saturation index. Correlation coefficients are shown in Table 2. *P < 0.05, **P < 0.01, and ***P < 0.001.

IMCLs had a significantly higher saturation index (CH_2_:CH_3_) in both muscles of the LD subjects compared with controls (SOL *P* = 0.008; TA *P* = 0.024) but not athletes (**Fig. 4A**, B). In the control group, smaller IMCL pools were associated with a higher saturation index, as shown by the linear regression line (dotted line in Fig. 3E, F) having a gradient (ΔCH_2_/ΔCH_3_) that was less than the mean CH_2_:CH_3_ (e.g., gradient SOL = 6.7 vs. mean CH_2_:CH_3_ = 8.8; gradient TA = 4.2 vs. mean CH_2_:CH_3_ = 6.0). This phenomenon seemed to be independent of insulin sensitivity in the controls (**Table 2**; there was no relation of HOMA-IR with IMCL concentration). To generate a pathophysiologically meaningful measure of composition that is independent of IMCL quantity, the vertical (CH_2_) deviation from this regression line was measured and taken as a marker of the saturation of the pool that is adjusted for quantity, which we term the adjusted saturation index (CH_2_:CH_3adj_). This adjusted compositional marker was significantly higher in LD subjects compared with athletes (SOL *P* = 0.001; TA *P* = 0.046) and controls (SOL *P* = 0.003), with a tendency in the TA that falls just short of conventional statistical significance (*P* = 0.06) (Fig. 4C, D).

**TABLE 2.** Correlation coefficients of whole-body insulin resistance with IMCLs

IMCL Measure	HOMA-IR			
	Controls (*n* = 38)	Controls and Athletes (*n* = 52)	Controls and LD Subjects (*n* = 54)*^a^*	Controls, LD Subjects, and Athletes (*n* = 68)*^b^*
SOL				
Concentration (CH_3_)	−0.016	0.248	0.149	0.312*
Concentration and composition (CH_2_)	0.153	0.315*	0.394**	0.477***
Composition (CH_2_:CH_3_)	0.217	−0.048	0.439***	0.241*
Composition adjusted for quantity (CH_2_:CH_3adj_)	0.320*	0.271*	0.583***	0.532***
TA				
Concentration (CH_3_)	0.200	0.251	0.205	0.258*
Concentration and composition (CH_2_)	0.224	0.247	0.338*	0.344**
Composition (CH_2_:CH_3_)	0.100	−0.046	0.337*	0.202
Composition adjusted for quantity (CH_2_:CH_3adj_)	0.218	0.127	0.445***	0.364**

*P < 0.05, **P < 0.01, and ***P ≤ 0.001.

a *n* = 50 for TA.

b *n* = 64 for TA.

Unlike the uncorrected measure of composition, this adjusted composition also had a significant relation to VO_2max_ (Fig. 4E, F) within the control subset alone, athletes alone (SOL), and control and athletes combined, such that fitter individuals had less saturated IMCLs for the same absolute quantity of IMCLs. VO_2max_ was significantly correlated with HOMA-IR in the control subset (*r* = −0.59, *P* = 0.003, *n* = 23) and in controls and athletes together (*r* = −0.53, *P* = 0.001, *n* = 37). Figure 4G and H show the relation of the adjusted composition to HOMA-IR. Table 2 shows relations of IMCL concentration and composition with insulin sensitivity. The saturation index was higher in the SOL muscle compared with the TA in all three groups. This was still the case in the two EMCL-deficient AGLD subjects (subject 1: SOL CH_2_:CH_3_ = 9.5 and TA CH_2_:CH_3_ = 6.3; subject 2: SOL CH_2_:CH_3_ = 11.2 and TA CH_2_:CH_3_ = 7.1). The IMCL relations with HOMA-IR were robust to differing fitting parameters, as shown in supplemental Table S1.

## DISCUSSION

Using a recently validated ^1^H MRS method we compared a compositional saturation index (CH_2_:CH_3_ ratio) of IMCLs in the SOL and TA muscles of female insulin-resistant LD subjects with that of age- and gender-matched athletes and healthy controls and showed it to be significantly higher in both muscles compared with controls but not athletes. The finding that smaller IMCL pools in the control group had a relatively higher saturation index than larger pools irrespective of insulin sensitivity could possibly explain why the athletes studied here, who had small IMCL pools, had a composition that was statistically similar to LD subjects. This observed concentration-composition relationship seems physiologically plausible given that more unsaturated and shorter-chain FAs are preferentially mobilized (17). A similar trend was also visible in a previous data set from both TA and SOL muscles of 19 healthy males after an 8 h fast (17). We hypothesize that a person may “move” along a line such as this while performing daily activities and that a measurement of deviation from this relationship may therefore be a more sensitive and specific measure of muscle metabolic physiology or pathophysiology. To take this concentration-composition dependence into consideration we adjusted the compositional saturation index for concentration (CH_2_:CH_3adj_), and this marker was able to distinguish between athletes and LD subjects in both muscles. The strong inverse relation of CH_2_:CH_3adj_ with VO_2max_ indicates that fitness is associated with a relatively lower saturation of IMCLs, although overall athletes and controls were not statistically different; this is similar to results from a biopsy study (29) that demonstrated a comparable percentage of saturated intramuscular triglycerides in male controls and athletes. Interestingly, previous reports of intramuscular triglyceride composition in insulin-resistant states (30, 31) yielded no difference in saturation percentage but did reveal a difference in linoleate (31). In these studies, the intramuscular triglyceride content was higher in insulin-resistant states; therefore, a higher saturation percentage may not be apparent given the composition-concentration relation we found. This lack of a difference in saturation could also equally be explained by the difference in sampling intramuscular versus intramyocellular pools, as EMCLs generally make up a significant proportion of intramuscular triglycerides (approximately twice the IMCL pool in our healthy cohorts even when voxels were placed to avoid visible marbling on T_1_-weighted images; Table 1).

By our measure of composition-independent IMCLs (using the CH_3_ instead of the uncorrected CH_2_ resonance), we found that the LD subjects’ IMCL concentration was not significantly higher in either muscle compared with age-, gender-, and BMI-matched controls but was higher in the SOL relative to athletes. There are few reports of IMCLs in LD subjects, but our findings are in agreement with Peterson et al. (32), who in three subjects with generalized lipodystrophy (two congenital and one acquired) found SOL IMCL concentrations that were similar to six age-, BMI-, and weight-matched controls. Calf IMCL concentration was also lower in a case report of acquired generalized lipodystrophy (33) compared with controls, while a study of four subjects with congenital generalized lipodystrophy suggested that IMCL concentration was higher (24). Using the CH_2_ resonance, as has been conventional in previous studies, we found SOL IMCL content to be significantly higher in our LD subjects compared with controls (Table 1), demonstrating, we argue, the influence of composition on measures of concentration.

### The athlete’s paradox

We found that the athletes’ IMCL concentration was significantly lower (SOL) or similar (TA) to controls. Although this seems to contradict well-known reports of an athlete’s paradox using both biopsy methods (13, 29, 34–36) and ^1^H MRS (37, 38), this finding is in agreement with studies that reported no such paradox compared with old or young controls (39), obese individuals (40), or in certain fiber types (14). It is known that athletes can have a large depletion-repletion range of IMCLs, and it is possible that the IMCL concentration had not fully recovered since the last training session (24–48 h prior), as IMCLs can still increase significantly after these intervals (41). In fact, our results demonstrate an inverse relation of IMCL content and VO_2max_ that is consistent with a study by Boesch et al. (42), in which a combination of daily training at 60% of the VO_2_ peak with a low-fat (10% to 15% fat) diet depleted IMCL levels in both the vastus lateralis and TA muscles to a consistent level that correlated with the VO_2_ peak, suggesting that our elite female athletes were nearly “empty” of IMCLs; we did not control for diet in our study.

### Relationship of whole-body insulin resistance to IMCLs

Within the controls, only the compositional saturation index adjusted for quantity (CH_2_:CH_3adj_) in the SOL was significantly correlated with whole-body insulin resistance (Table 2). The inclusion of insulin-resistant LD subjects increases the statistical significance of this relation and yields associations with other composition-influenced markers, but not IMCL concentration, in either muscle. In our study, the addition of athletes predictively reduced the associations with composition, as their pools were small and therefore had a tendency for a higher saturation index, but relations remained with measures that reflect large saturated pools (i.e., CH_2_:CH_3adj_ and CH_2_). These striking results suggest that the accumulation of saturated IMCLs and not concentration alone relates to early-stage insulin resistance.

Supporting our findings, the concentration of the most abundant saturated fat, palmitic acid, within muscle triglycerides has been shown to be related to insulin sensitivity (43). Palmitic acid is known to increase ceramide concentrations, which are thought to engage stress-responsive serine kinases that impede insulin activation of its cell-surface receptor, as well as downstream signaling molecules such as insulin receptor substrate 1 and protein kinase B/Akt (44). Our study was of course not able to probe these mechanisms.

### Limitations

Unlike previous ^1^H MRS studies that have conventionally reported IMCL concentrations using the predominant CH_2_ resonance while assuming a notional normal composition, here we utilized the smaller CH_3_ resonance, which has the advantage of composition independence and, with comprehensive prior-knowledge constraints, including line-width constraints relative to the CH_2_ resonance (22), fitted the IMCL CH_3_ resonance from the overlapping EMCL/IMCL CH_3_ signals. Due to fiber-orientation differences between the SOL and TA muscles, the EMCL resonances are very slightly systematically shifted between muscle groups. Despite this potential for a systematic difference in the CH_2_:CH_3_ ratio between muscles, this ratio was still higher in the SOL compared with the TA muscle in both EMCL-deficient AGLD subjects, consistent with findings in our other participants in this study and a previous study (17) suggesting a compositional difference between muscles. Our LD subjects, who mainly had partial forms of lipodystrophy, had overall similar quantities of EMCLs to those of our controls and athletes that has helped to reduce potential intergroup influence of this overlapping resonance. In addition, the relations of IMCLs with HOMA-IR were robust to differing fitting routines, including constraining the EMCL CH_3_ amplitude and accounting for asymmetric line shapes. These findings, together with the finding that neither the IMCL CH_2_:CH_3_ nor IMCL CH_2_:CH_3adj_ markers related to either EMCL CH_2_ or CH_3_, suggests a lack of EMCL influence in our data sets; however, it is possible that in other insulin-resistant cohorts large overlapping EMCL resonances may be a confounding factor.

### Summary

The use of our recently validated and potentially widely applicable ^1^H MRS approach to determine both the IMCL composition and concentration independent of composition within the SOL and TA muscles of female individuals covering a wide range of insulin sensitivities has revealed that markers of the accumulation of saturated triglycerides in the IMCL pool are more strongly associated with whole-body insulin resistance than IMCL concentration alone. Differences in associations of insulin resistance with IMCL concentration when using the CH_3_ and conventional CH_2_ peaks for quantification highlights the need for awareness of the potential influence of composition on previously reported ^1^H MRS measures of concentration.

Our finding of a strong relationship between VO_2max_ and relatively unsaturated IMCL pools in controls and athletes points to a role of exercise in decreasing the amount of saturated fat within the IMCL store. The association of insulin resistance with the accumulation of saturated IMCLs, even within a healthy control population, could suggest an early involvement in its pathogenesis and provide a reason why combined exercise and diet are effective therapeutic options in the early stages of insulin resistance.

## Supplementary Material

Supplemental Data
