# Supplementary material for: Accumulation of saturated intramyocellular lipid is associated with insulin resistance
Source: J Lipid Res. 2019 May 2;60(7):1323–32. doi: 10.1194/jlr.M091942 (PMC6602127; doi:10.1194/jlr.M091942)
Supplement: Supplemental Data [file supp_60_7_1323__index.html]

Accumulation of saturated intramyocellular lipid is associated with insulin resistance — Accumulation of saturated intramyocellular lipid is associated with insulin resistance — Supplemental Data 

# Accumulation of saturated intramyocellular lipid is associated with insulin resistance

## Supplemental Data

- Supplemental Figure S1; Supplemental Table S1 (.docx, 82 KB) - Supplemental Figure S1. Soleus IMCL composition-independent concentration is significantly lower (p = 0.025) in athletes (white bar, n = 14) compared with a subset of the controls that are percentage body fat matched (grey bar, n = 7). Supplemental Table S1. Summary of different spectral fitting routine parameters (upper), results from lipodystrophic patients and controls combined (middle), and their relations to HOMA-IR (lower).
