# Supplementary material for: Accumulation of saturated intramyocellular lipid is associated with insulin resistance
Source: J Lipid Res. 2019 May 2;60(7):1323–32. doi: 10.1194/jlr.M091942 (PMC6602127; doi:10.1194/jlr.M091942)
Supplement: Supplemental Data [file 10.1194_M091942_jlr.M091942-1.docx]

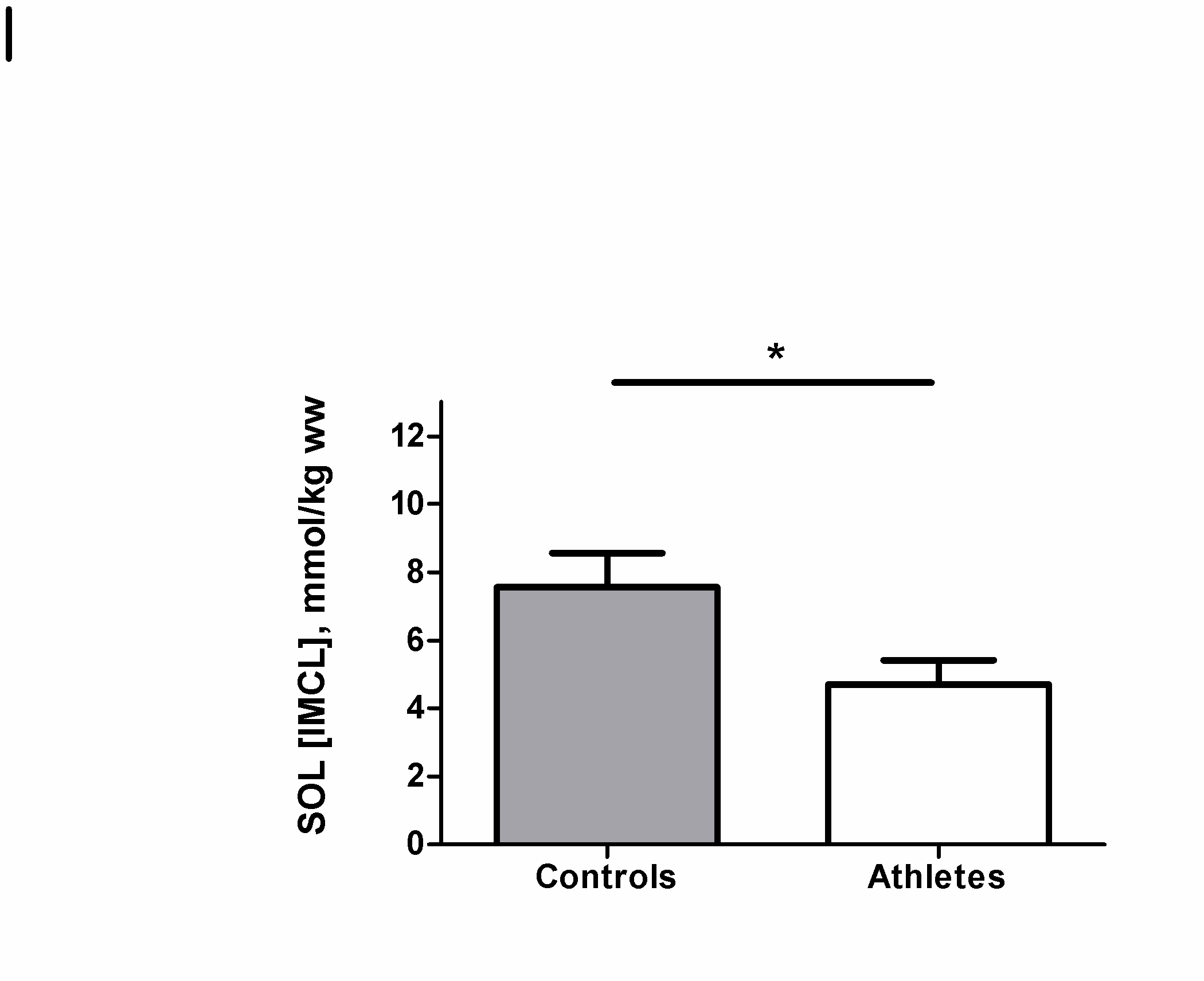


**Supplemental Figure S1.** Soleus IMCL composition-independent concentration is significantly lower (p = 0.025) in athletes (white bar, n = 14) compared with a subset of the controls that are percentage body fat matched (grey bar, n = 7).

**Supplemental Table S1.** Summary of different spectral fitting routine parameters (*upper*), results from lipodystrophic patients and controls combined (*middle*), and their relations to HOMA-IR (*lower*).

|  | **Fitting routine** | | | | |
| --- | --- | --- | --- | --- | --- |
|  | **1** | **2** | **3** | **4** | |
| **Fitting parameters**  No. of EMCL lineshapes | 1 | 1 | 2 | 1 | |
| LW EMCL CH_3_  LW IMCL CH_3_ | E2 + α  I2 + α | E2 + α  I2 + α | E2_a_ + α, E2_b_ + α  I2 + α | Fit 1-30 Hz  Fit 1-30 Hz | |
| Frequency EMCL CH_3_  Frequency IMCL CH_3_ | E2 - c  I2 - c | E2 - c  I2 - c | E2_a_ - c, E2_b_ - c  I2 - c | E2 - c  I2 - c | |
| Resonance area EMCL CH_3_  Resonance area IMCL CH_3_ | Free  Free | E2 * k  Free | E2_a_ * k, E2_b_ * k  Free | Free  Free | |
| **Fitted results**  *Soleus* |  |  |  | |  |
| IMCL CH_3_ | 0.18±0.01 | 0.21±0.01 | 0.22±0.18 | | 0.19±0.01 |
| IMCL CH_2_ | 1.56±0.10 | 1.64±0.10 | 1.66±0.11 | | 1.56±0.10 |
| EMCL CH_3_ | 0.43±0.03 | 0.23±0.02 | 0.23±0.02 | | 0.44±0.03 |
| EMCL CH_2_ | 1.99±0.15 | 2.00±0.16 | 1.99±0.15 | | 1.92±0.15 |
| *Tibialis Anterior* |  |  |  | |  |
| IMCL CH_3_ | 0.12±0.01 | 0.13±0.01 | 0.13±0.01 | | 0.14±0.01 |
| IMCL CH_2_ | 0.72±0.05 | 0.75±0.05 | 0.73±0.05 | | 0.70±0.05 |
| EMCL CH_3_ | 0.29±0.02 | 0.23±0.02 | 0.24±0.02 | | 0.29±0.02 |
| EMCL CH_2_ | 1.99±0.16 | 1.99±0.16 | 2.10±0.18 | | 2.00±0.16 |
| **Correlation coefficients with HOMA-IR**  *Soleus* |  |  |  | |  |
| IMCL CH_3_ | 0.149 | 0.115 | 0.184 | | 0.154 |
| IMCL CH_2_ | 0.394** | 0.287* | 0.350* | | 0.316* |
| IMCL CH_2_:CH_3_ | 0.439*** | 0.435*** | 0.376** | | 0.260 ^§^ |
| IMCL CH_2_:CH_3adj_ | 0.583*** | 0.578*** | 0.559*** | | 0.398** |
| *Tibialis Anterior* |  |  |  | |  |
| IMCL CH_3_ | 0.205 | 0.032 | 0.150 | | 0.076 |
| IMCL CH_2_ | 0.338* | 0.223 | 0.329* | | 0.212 |
| IMCL CH_2_:CH_3_ | 0.337* | 0.305* | 0.314* | | 0.245 ^§^ |
| IMCL CH_2_:CH_3adj_ | 0.445*** | 0.301* | 0.343* | | 0.244 ^§^ |

IMCL, intramyocellular lipid; EMCL, extramyocellular lipid; CH_3_, methyl protons resonating at 0.9 ppm; CH_2_, methylene protons resonating at 1.3 ppm. E2, EMCL CH_2_; I2, IMCL CH_2_; E2_a_, Gaussian 1 of EMCL CH_2_; E2_b_, Gaussian 2 of EMCL CH_2_; α, a value of approximately 2.5 Hz; c, a constant of 50.4 Hz; k, a constant of 0.1136. Routine 1 is the original fitting as described in the Methods section and previously used in (1). Routine 2 fixes the EMCL CH_3_ amplitude relative to the CH_2_ amplitude. Routine 3 allows two Gaussian lineshapes to fit the EMCL resonances to account for asymmetric EMCL lineshapes. The EMCL CH_3_ frequency, linewidth and amplitude were set relative to the CH_2_ resonance thus yielding similar CH_2_ and CH_3_ EMCL lineshapes. Routine 4 provides the most freedom by allowing the fitting of both EMCL and IMCL CH_3_ linewidths and amplitudes. Results are mean ± SEM. SOL n=54, TA n=50. HOMA-IR, Homeostasis Model Assessment of Insulin Resistance; CH_2_:CH_3_, compositional saturation index calculated as the ratio of CH_2_ to CH_3_ resonances; CH_2_:CH_3adj_, CH_2_:CH_3_ saturation index adjusted for lipid quantity. *p<0.05, **p<0.01, ***p≤0.001 (§ p<0.1).
